# Distant metastasis without regional progression in non-muscle invasive bladder cancer: case report and pooled analysis of literature

**DOI:** 10.1186/s12957-022-02664-5

**Published:** 2022-07-06

**Authors:** Tianyuan Xu, Wenyu Gu, Xianjin Wang, Leilei Xia, Yanyan He, Fan Dong, Bin Yang, Xudong Yao

**Affiliations:** 1 Department of Urology, Shanghai Tenth People’s Hospital, Tongji University, Shanghai, China; 2 Institute of Urinary Oncology, Tongji University School of Medicine, Shanghai, China; 3 Department of Urology, Ruijin Hospital, Shanghai Jiaotong University School of Medicine, Shanghai, China; 4 Division of Urology, Department of Surgery, University of Pennsylvania Perelman School of Medicine, Philadelphia, PA USA; 5 Department of Pathology, Shanghai Tenth People’s Hospital, Tongji University, Shanghai, China; 6 Department of Reproductive Medicine, Renji Hospital, Shanghai Jiaotong University School of Medicine, Shanghai, China

**Keywords:** Non-muscle invasive bladder cancer, Metastasis, Metastasectomy, Chemotherapy, Immunotherapy, Radiotherapy

## Abstract

**Background:**

Non-muscle invasive bladder cancer (NMIBC) represents the majority of bladder neoplasms. It is unusual for NMIBC metastasizing distantly without regional progression, namely metastatic NMIBC (mNMIBC), which is still poorly understood and easily omitted based on current management policies. So far, description of mNMIBC is limited to a few case reports.

**Methods:**

We reported a 70-year-old man with NMIBC who suffered from cervical metastasis without pelvic recurrence at 41 months after initial diagnosis. Then we performed a collective analysis of this case together with published mNMIBC cases searched from PubMed, Embase, and Web of Science, aiming to illustrate baseline clinicopathologic parameters, metastatic patterns, and treatment outcomes of these patients and analyze associated influencing factors.

**Results:**

After scrupulous review, 45 cases previous reported and the one from our center were incorporated into the aggregated cohort of mNMIBC, including 34 males and 12 females. Primary tumors from 46.7% of patients were high-grade (HG) or grade 3 (G3) and 65.1% had T1 lesions. Aberrant biomarker expression was found in tumors of some cases. Most (40/46) metastases of mNMIBC occurred at a single site, mainly in lung, bone and lymph nodes. Apart from three cases of de novo mNMIBC, the mean metastasis-free survival (MFS) interval of metachronous mNMIBC was 42.5 months, which was obviously longer than conventional metastatic bladder cancer. Shortened MFS interval was associated with old age, T1 or HG/G3 primary tumors, and non-lung metastases. Systemic chemotherapy and metastasectomy or radiotherapy for oligometastatic lesion were main therapeutic approaches of mNMIBC, and immunotherapy was adopted for the case from our center. Lung and bone metastases correlated with relatively favorable and unfavorable survival outcomes, respectively. Compared with monotherapy, chemotherapy, or immunotherapy combined with local cytoreduction got more favorable outcomes.

**Conclusion:**

Although rare, mNMIBC occurs more in tumors with high-risk features. Usually, mNMIBC metastasizes later than conventional metastatic bladder cancer and manifests as solitary lesion. Outcomes of mNMIBC would be influenced by metastatic site and post-metastatic treatment. Systemic treatment combined with local cytoreduction may render survival benefit in selected patients.

**Supplementary Information:**

The online version contains supplementary material available at 10.1186/s12957-022-02664-5.

## Background

With estimated 573,278 new cases every year, bladder cancer ranks as the second most common genitourinary malignancy around the world and most are classified as urothelial carcinomas [1]. Among newly diagnosed cases, 70% and 30% manifest as non-muscle invasive bladder cancer (NMIBC) and muscle-invasive (MIBC) or metastatic bladder cancer (mBC), respectively. Surgical treatments of NMIBC include transurethral resection of bladder tumor (TURBT) with electrical or laser equipment and re-staging resection for some high-risk cases [2–4]. However, over half of NMIBC lesions still recur, including the minority which progress to higher-stage disease. Radical cystectomy is recommended for MIBC, but 50% will still relapse locally or metastasize distantly. Besides, 10–15% of patients are already metastatic at initial diagnosis and the survival rarely exceeds 3–6 months if without effective treatment [5]. Locally or distantly metastasis is not unusual, but generally observed in those with MIBC.

Although the natural history of most NMIBC is to remain “non-invasive”, tendency for progression to MIBC is substantial at 10–20%. Less than 5% of NMIBC might also relapse as regional metastatic lymph nodes without muscle invasion [6]. However, it is rare for NMIBC to metastasize directly to distant sites without progression in bladder or regional lymph nodes. Matthew et al. [7] reviewed over 1000 cases of bladder cancer and identified five who had NMIBCs whereas subsequently suffered from distant metastases, namely metastatic NMIBC (mNMIBC). Owing to the scarcity of such cases, only a few isolated case reports were published. Current NMIBC follow-up policies put most of attention on cystoscopy and pelvic or urologic imaging, but scarcely any on distant sites like chest, cervical region, craniocerebrum, and limbs. It is still unclear about baseline characteristics, metastatic patterns, therapeutic approaches, and outcomes of the mNMIBC population.

Here, we report a case of mNMIBC who acquired sustained response after multimodal therapy at our center. For the first time, we also performed collective analysis on a medium sample cohort of mNMIBC cases aggregated from our experience together with previous reports, which may help find some characteristics of and hints for the optimal care of these patients.

## Methods

### Report of the case from our center

With approval from the institutional ethical committee, mNMIBC cases were searched from electronic medical record database of Shanghai Tenth People’s Hospital and one patient was identified. The patient had given his informed consent for the anonymous use of their personal data and further publication when admission to our department. Detailed report of this case is presented below.

### Literature search and case selection

In May 2022, comprehensive computerized literature search of PubMed, Embase, and Web of Science in the field of mNMIBC was performed. Search terms were “non-muscle invasive,” “nonmuscle invasive,” “Ta,” “T1,” “superficial,” and “bladder cancer,” “bladder carcinoma,” and “metastasis,” “metastases,” for potentially eligible studies. No language restriction was used. Additional manual searches were performed of reference lists from included studies. Literatures without mentioning metastasis or NMIBC were removed by screening titles and abstracts. Included studies were reviewed to screen cases with non-urothelial tumor, non-NMIBC disease (i.e., MIBC, malignancy in upper urinary tract) or progression to pelvic lymph nodes. NMIBC cohorts or individuals receiving cystectomy were not included. Cases without information of sex or age or any pathologic description about the primary tumor were also excluded.

### Data extraction

We performed a collective analysis of eligible published mNMIBC cases together with the one from our center. Demographic and clinicopathologic data were collected and analyzed if available, including patient sex, age, tumor size, number, and pathologic parameters at initial diagnosis, times of intravesical recurrences, interval from initial diagnosis to metastasis (metastasis-free survival, MFS), metastatic site, further treatment, and outcomes. Pathologic stage was assessed according to the TNM staging system, with NMIBC categorized as Ta, T1, or carcinoma in situ (CIS), and tumor grade was determined according to 1973 or 2004 WHO systems as described in each included study, namely grade 1–3 (G1–3) or papillary urothelial neoplasm of low malignant potential, low-grade (LG), and high-grade (HG) [8]. Biomarker expression of tumor tissue, as detected by immunohistochemistry (IHC) staining, was also recorded if available. As for cases of our center, primary tumor specimens were processed and evaluated by urologic pathologist, using both hematoxylin-eosin and IHC staining.

### Statistical analyses

Statistical analyses were performed by SPSS 23.0 software (IBM Corporation, Armonk, NY, USA). One-way ANOVA and *χ*2 or Fisher’s test were used to compare quantitative data and categorical data, respectively. Survival curves were estimated using Kaplan-Meier method and log-rank test was used to assess difference significance. Lollipop chart and forest plots were drawn using R 3.4.2 software (http://www.r-project.org/). In all tests, two sides of *P* < 0.05 were considered statistically significant.

## Results

### Case presentation

**Fig. 1:**
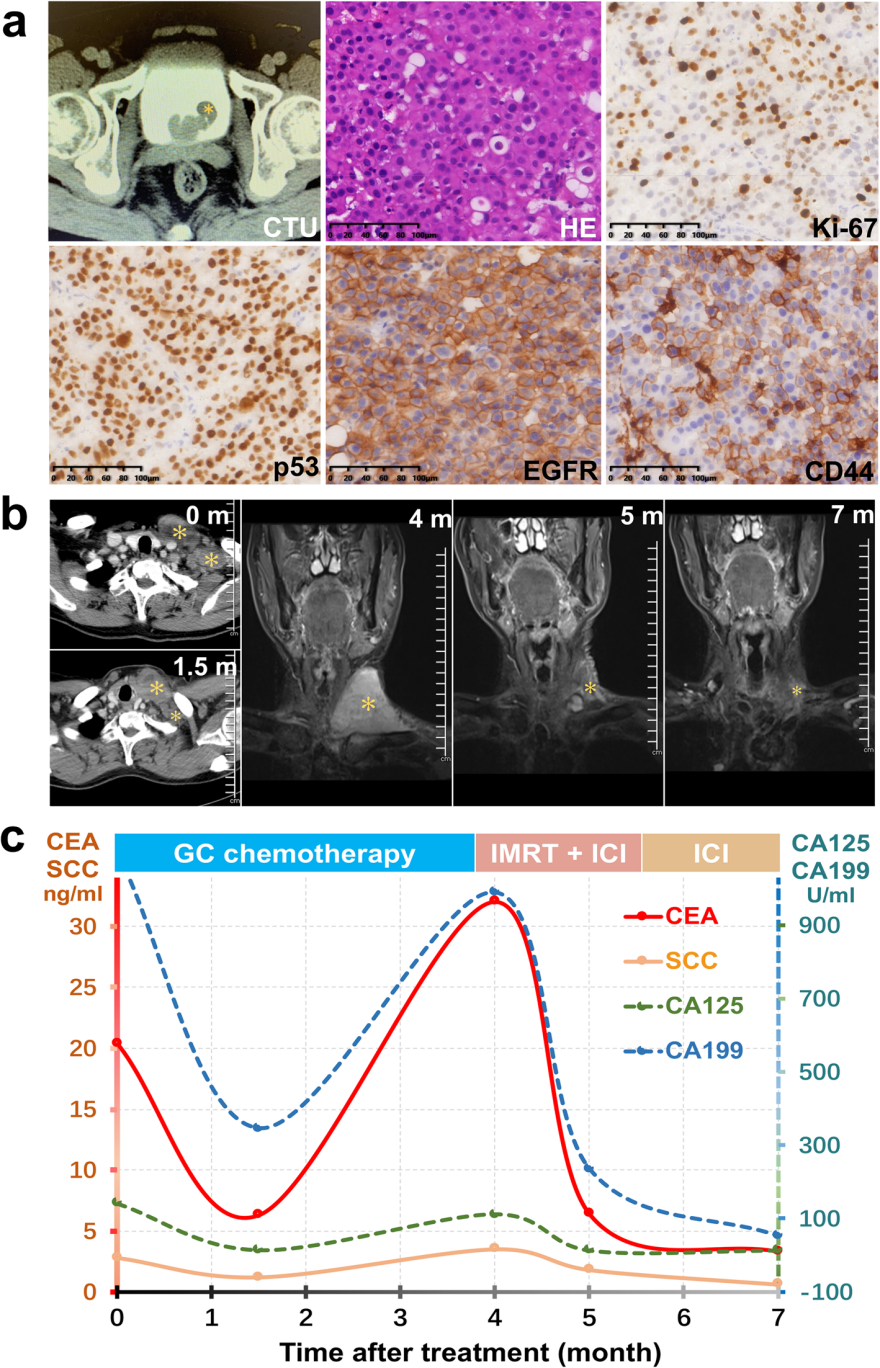
Presentation of the mNMIBC case from our center: CT urography and pathologic findings of primary tumor (**a**); dynamic changes of cervical lesion in CT/MR scans (**b**) and fluctuating levels of serum tumor markers (**c**) during post-metastatic treatment (yellow asterisk referring to primary or metastatic lesions)

A 70-year-old male was admitted into our center with gross hematuria and CT urography found a 3-cm bladder neoplasm. Neither regional nor distant metastasis was detected. A solitary cauliflower-like tumor at left bladder neck accompanied with muscle tissues were resected by TURBT and T1HG urothelial carcinoma was confirmed. IHC analyses showed widespread nuclear expressions of Ki-67 and p53 as well as strong and diffuse expression of epidermal growth factor receptor (EGFR) and CD44 (Fig. 1a). Since the patient showed intolerance to bacillus Calmette-Guérin (BCG), intravesical instillations of epirubicin were postoperatively administered for 18 months. Regular follow-ups showed free of disease until 41 months after TURBT, when he complained about a left cervical mass. The mass was gradually enlarged and biopsy was performed 5 months later, confirming lymph node metastasis of urothelial carcinoma. Cystoscopy and systematic radiographic examinations revealed no regional relapse or other metastasis. Chemotherapy was initiated with gemcitabine-cisplatin regimen. Assessment at cycle 3 showed significant shrink of the cervical mass and declined serum tumor markers of carcinoma embryonic antigen (CEA), carbohydrate antigen-199 (CA199), CA125, and squamous cell carcinoma antigen (SCC) (Fig. 1b, c).

However, the lesion then grew in size and elevated serum tumor markers were detected at cycle 5. Sample of metastatic lesion was subjected to next-generation sequencing (NGS) using a 642-gene panel (supplementary material Table S1). Gene variants are summarized in Table 1. Tumor mutational burden (TMB) was 20.22 mutations/Mb and germline frameshift mutation of CHEK2 was detected. Main somatic alterations involved truncating mutations in KDM6A, ARID1A, CDH1, and missense mutations in PIK3CA, ERCC2. Amplified and deleted genes included EGFR, MYCN and CDKN2A, CDKN2B, respectively. The promoter of TERT harbored a point mutation and somatic gene fusion of FGFR3-TACC3 was also found. Intensity-modulated radiotherapy (IMRT) of cervical tumor-draining lymph nodes was then administered combined with immune checkpoint inhibition (ICI) of off-label sintilimab, which is the first approved programmed death 1 inhibitor in China with affordable price. After 1 month, the treatment yielded remarkable response, as shown by MR imaging and serum tumor marker tests. Sustained remission was observed during further sintilimab monotherapy (Fig. 1b, c) and the patient had stable disease at 1.6 years’ follow-up after metastasis.

**Table 1:** Gene variants in metastatic tissues of the case from our center

Gene	Variant	Amino acid change	Abundance/copy	Variant type
CHEK2	c.472delA	p.I158fs	Heterozygous	Frameshift (germline)
GATA1	c.907C>G	p.Q303E	79.40%	Missense
TERT	c.-124C>T	–	71.00%	Promoter mutation
STAG2	c.1408G>T	p.E470X	65.30%	Nonsense
KDM6A	c.3361_3384delinsT	p.H1121fs	60.00%	Frameshift
ERCC2	c.713A>G	p.N238S	59.40%	Missense
ARID1A	c.2183_2208del26	p.P728fs	53.30%	Frameshift
PIK3CA	c.1637A>C	p.Q546P	30.30%	Missense
CDH1	c.2222_2250del29	p.L741fs	22.90%	Frameshift
INHBA	c.362C>T	p.S121L	49.90%	Missense
SF3B1	c.961G>T	p.D321Y	49.50%	Missense
SPEN	c.1604G>A	p.R535Q	47.90%	Missense
NOTCH3	c.539C>T	p.S180F	47.70%	Missense
PCBP1	c.910G>A	p.E304K	42.60%	Missense
FAT4	c.760C>T	p.H254Y	39.00%	Missense
IRS2	c.379G>A	p.E127K	35.70%	Missense
IPO7	c.1957G>A	p.E653K	33.60%	Missense
ACVR2A	c.821C>T	p.S274L	32.60%	Missense
CDH1	c.1613A>G	p.D538G	32.20%	Missense
CDK12	c.2629G>A	p.D877N	32.20%	Missense
TBX3	c.1285G>A	p.D429N	26.60%	Missense
ERCC3	c.1274G>A	p.R425Q	26.30%	Missense
SF3B1	c.2704G>A	p.E902K	26.00%	Missense
VTCN1	c.458C>G	p.P153R	25.50%	Missense
KIF5B	c.2208A>C	p.Q736H	25.00%	Missense
NOTCH3	c.1636G>T	p.V546L	24.90%	Missense
TXNDC8	c.109A>G	p.R37G	24.00%	Missense
MALT1	c.955G>A	p.E319K	23.90%	Missense
BICC1	c.2698G>A	p.D900N	23.30%	Missense
MYCL	c.694C>T	p.H232Y	23.20%	Missense
ZNF521	c.680A>G	p.N227S	23.10%	Missense
PTPRK	c.1603G>C	p.D535H	21.90%	Missense
TACC3	c.772G>A	p.E258K	21.00%	Missense
PREX2	c.3079C>T	p.Q1027X	19.00%	Nonsense
MUC17	c.6295G>A	p.E2099K	16.70%	Missense
KMT2C	c.871C>G	p.L291V	14.50%	Missense
DDX5	c.907G>A	p.G303S	14.00%	Missense
INHBA	c.485C>T	p.P162L	13.30%	Missense
AR	c.179A>T	p.Q60L	1.80%	Missense
CDK12	c.3494C>T	p.S1165F	1.70%	Missense
ATRX	c.3875C>T	p.S1292L	1.30%	Missense
FGFR3	FGFR3-TACC3	–	9.02%	Gene fusion
EGFR	–	–	1.72	Amplification
MYCN	–	–	1.61	Amplification
AR	–	–	1.56	Amplification
FGFR1	–	–	1.55	Amplification
CDKN2A	–	–	0.30	Deletion
CDKN2B	–	–	0.30	Deletion

### Baseline clinicopathological features of the pooled mNMIBC cohort

**Fig. 2:**
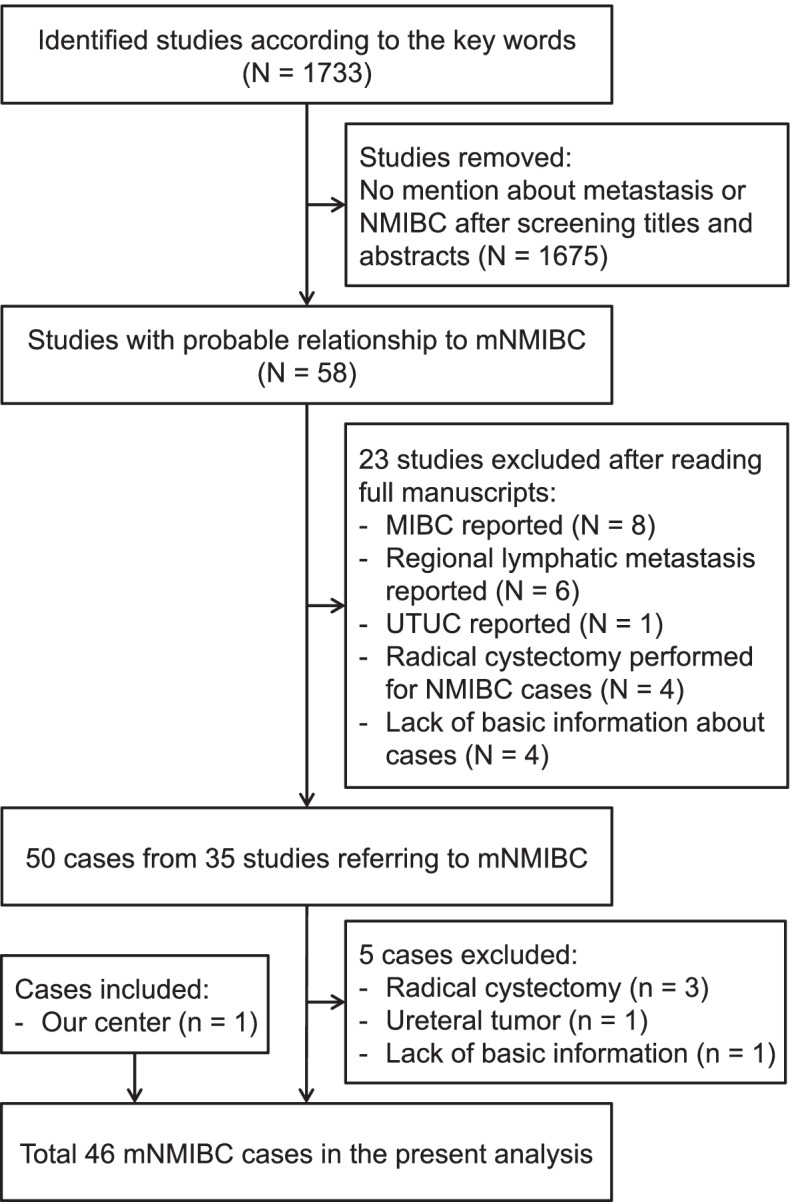
Flow chart of study selection and case inclusion

The process of study selection and case inclusion is depicted in Fig. 2. Initial literature search found 1733 potential studies, among which 1698 were excluded after reading titles, abstracts or manuscripts. Finally, 45 cases from 35 reports (listed in supplementary material) met the criteria of mNMIBC for collective analysis, including 20 from east Asia, 11 from Europe, 11 from the USA, and 3 from west Asia. Thus, the aggregated cohort comprised totally 46 mNMIBC patients, including the one from our center.

Table 2 shows detailed data of all cases, including 34 males and 12 females. Baseline and metastatic features of the cohort are summarized in Table 3. At initial diagnosis, their mean age was 64.1 years (range 33–95) and all received TURBT or partial cystectomy. HG/G3 tumors were found in 21 patients and other 24 had LG/G1-2 disease. Pathologic stage analysis within 43 cases showed 30.2% of Ta, 65.1% of T1 and 14% of CIS (2 of pure CIS, 4 of T1 concomitant with CIS). Besides, variant histology, lymphovascular invasion (LVI), or aberrant expressions of IHC markers were reported in primary tumors of eight cases, including the one from our center. Morphologic information of initial lesions was provided for the case from our center and 27 counterparts from 18 studies. Multiple and large (diameter ≥ 3 cm) tumors were observed in 52% and 62% of available cases, respectively.

**Table 2:** Detailed data of mNMIBC cases from identified studies (see supplementary material) and our center

Case	Study	Age, year	Sex	Characteristics of the initial tumor			Times of relapses	MFS interval, months	Sites of metastases	Post-metastatic treatment	Outcomes
				Size, cm	No.	Pathology					
1	Seymour, 1972	33	M	> 3	1	G2	0	117	Lung	ROM	NED after 1 year
2	Matthews, 1984	35	F	/	≥ 2	T1G2	≥ 2	86	Bone	/	DOD after 6 months
3	Matthews, 1984	48	F	/	≥ 2	TaG1	≥ 2	100	Lung	/	/
4	Matthews, 1984	76	M	/	1	TaG2	2	14	Bone	/	DOD after 1 months
5	Matthews, 1984	73	M	/	2	T1G2	0	25	Lung	/	DOD after 1 months
6	Matthews, 1984	57	M	/	≥ 2	T1G3	≥ 2	70	Liver	/	DOD after 2 months
7	Matthews, 1984	77	M	/	≥ 2	T1G2	0	12	Lung	/	/
8	Andriole, 1985	60	F	/	/	T1G1	5	31	Ovary, tube, uterus	ROM, CT	Local progression after 31 months
9	Francis, 1992	70	F	/	/	TaG1	≥ 2	84	Ovary	ROM	/
10	Kakehi, 1992	63	M	> 3	> 5	TaG2	5	46	Lung	/	/
11	Kakehi, 1992	55	M	< 1	2	T1G2	0	26	Inguinal LN	/	/
12	Kakehi, 1992	48	M	> 3	> 5	T1G3	3	12	Inguinal LN	/	/
13	Kakehi, 1992	51	M	1–3	> 5	TaG2	5	38	Lung	ROM	/
14	Kawashima, 1993	67	M	> 3	≥ 2	T1G3	2	/	Bone	/	DOD
15	Koh, 1994	33	M	/	/	TaG1	≥ 1	108	Lung	CT, ROM	NED after 14 months
16	Kardar, 1998	60	F	/	/	TaG2	3	48	Ovary	ROM	DOD after 3 months
17	Saito, 1999	79	M	/	/	T1G2	0	18	Skin	ROM, CT	NED after 15 months
18	Davies, 2003	56	M	2	1	T1HG, p63(+)	/	0	Brain	ROM RT, CT	/
19	Shikishima, 2006	71	M	/	/	T1G2	2	36	Orbit, bone	RT, CT	DOD after 7 months
20	Hirayama, 2007	64	F	4	≥ 2	T1G1	4	30	Lung	ROM, IVC	NED after 6 years
21	Murakami, 2007	76	F	4	1	T1G3	0	23	Uterus	RT	PR after 4 months
22	Haga, 2008	95	M	/	/	T1G3	1	9	Lung	None	DOD after 1 months
23	Zennami, 2008	65	M	2	2	T1G3, CIS, HER-2(+)	0	34	Brain	ROM	DOD after 2.5 months
24	Dougherty, 2009	66	M	/	/	LG	≥ 2	120	Lung	ROM, CT	NED after 1 year
25	Blasberg, 2009	83	F	/	/	T1HG, sarcomatoid	0	3	Colon	ROM	/
26	D’Souza, 2011	69	F	2	1	T1G3, LVI(+)	0	10	Cerebellum	RT	NED after 21 months
27	Arai, 2012	52	F	3	1	T1G3	2	19	Lung	CT, ROM	NED after 53 months
28	Canter, 2012	76	F	3	1	T1HG, CIS, micropapillary	/	0	Pancreas	CT	/
29	Madan, 2012	85	M	/	/	LG	1	12	Lung	RT	/
30	Sasaki, 2013	66	M	3.8	3	T1HG, CIS	1	10	Bone	CT	PR after 6 months
31	Sano, 2013	60	M	< 1	/	TaLG	6	60	Lung	ROM, CT	NED after 30 months
32	Zalawadia, 2014	65	F	/	/	T1	0	60	Liver	None	DOD within 1 week
33	Hong, 2015	60	M	4.5	1	T1HG, ki67(70%+), p53(70%+), EGFR(+)	0	3	Bone	CT	DOD after 4 months
34	Vural, 2015	53	M	/	1	TaLG	3	96	Lung	CT, ROM	/
35	Kelten, 2015	75	M	3	1	T1HG, CIS	0	15	Cerebellum	ROM, RT	DOD after 2 months
36	Teyssonneau, 2017	60	M	/	/	T1HG	0	36	Meninges, bone	CT	NED after 4 years
37	Kida, 2018	77	M	2	1	T1HG, LVI (+)	0	10	Bone, liver	RT	DOD after 5 months
38	Kida, 2018	70	M	2	1	T1HG	0	18	Retroperitoneal LN	ROM, CT	NED after 1 year
39	Frydenlund, 2018	52	M	3	3	TaHG	0	30	Bone	RT, CT	/
40	Juri, 2018	63	M	/	/	CIS, HG	0	60	Cervical LN	/	/
41	Juri, 2018	79	M	/	/	CIS, HG	3	63	Mediastinal LN, bone	/	/
42	Rodríguez-López, 2018	58	M	/	/	TaLG	6	> 60	Lung	ROM, CT	NED after 3 years
43	Garrido-Abad, 2019	67	M	/	/	TaLG	1	60	Cervical LN	CT	DOD after 5 months
44	Defant, 2020	64	M	/	/	TaLG	/	0	Bone	ROM, CT, RT	NED after 11 years
45	Nishiyama, 2021	68	M	/	/	T1G2	0	51	Testis, para-aortic LN, peritoneum	ROM, CT	NED after 1.3 years
**46**	***Xu, 2022***	70	M	3	1	T1HG, ki67(65%+), p53(+), p63(+), EGFR(+), CD44(+)	0	41	Cervical LN	CT, ICI, RT	PR after 1.6 years

*CIS* carcinoma in situ, *CT* chemotherapy, *DOD* dead of disease, *HG* high grade, *ICI* immune checkpoint inhibition, *IVC* intravesical chemotherapy, *LG* low grade, *LN* lymph nodes, *LVI* lymphovascular invasion, *MFS* metastasis-free survival, *NED* no evidence of disease, *PR* partial remission, *ROM* resection of metastases, *RT* radiotherapy

**Table 3:** Baseline clinicopathologic and metastatic characteristics of the mNMIBC cohort

Characteristics	No.	%
Age, years		
< 70	30	65.2
≥ 70	16	34.8
Gender		
Male	34	73.9
Female	12	26.1
Tumor stage		
Ta	13	30.2
T1	28	65.1
CIS	6	14.0
Tumor grade		
G1-2/LG	24	53.3
G3/HG	21	46.7
Tumor size, cm		
< 3	8	38.1
≥ 3	13	61.9
Number of tumors		
Single	13	48.1
Multiple	14	51.9
Metastatic sites		
Lung	15	32.6
Bone	11	23.9
Lymph nodes	8	17.4
Liver	3	6.5
Central nervous system	5	10.9
Genital system	5	10.9
Others	5	10.9

*CIS* carcinoma in situ, *HG* high grade, *LG* low grade

### Patterns and characteristics of distant metastases

**Fig. 3:**
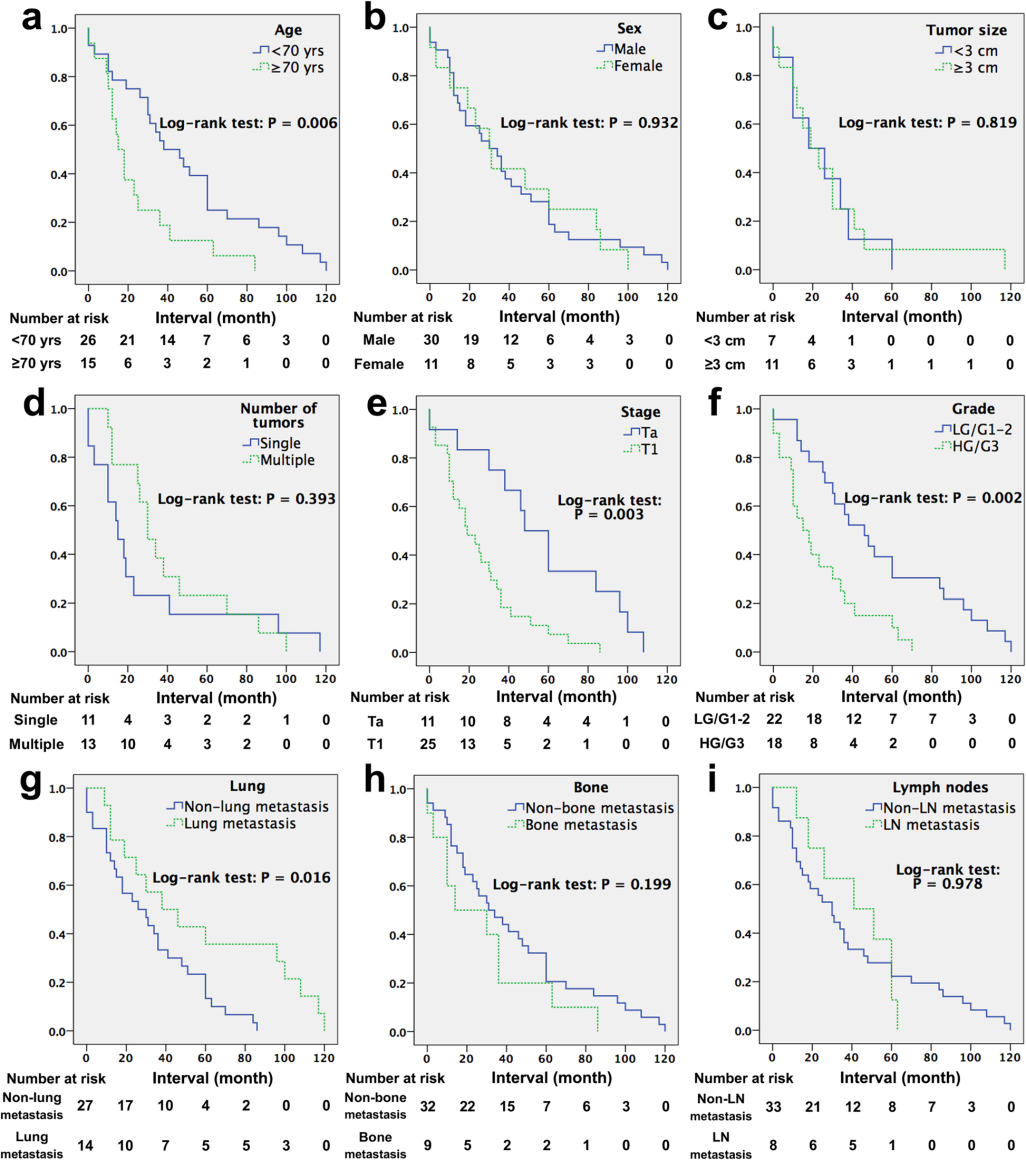
Metastasis-free survival curves of mNMIBC cases, stratified according to patient age (**a**), sex (**b**), tumor size (**c**), number (**d**), stage (**e**) and grade (**f**) at initial diagnosis as well as whether metastases occurring in lung (**g**), bone (**h**), or lymph node (**i**)

Mean duration from initial finding of NMIBC to confirmation of metastasis, namely MFS interval, was 42.5 months (range 3–120). During this period, NMIBC recurred in 56% of patients, with an average of ≥ 2.7 times. Besides, another three patients were initially found NMIBC and metastasis simultaneously. For all mNMIBC cases, metastatic sites included lung (15), bone (11), central nervous system (5), female genital system (4), liver (3), pancreas (1), colon (1), skin (1), orbit (1), testis (1), peritoneum (1), and distant lymph nodes (8). Four patients had two organs with metastases, one male had para-aortic lymphatic, peritoneal, and bilateral testicular metastases, and another female had lesions involving ovary, tube, and uterus. Beyond these patients, all others had a single-site metastasis (Table 2). Kaplan–Meier analyses were employed to determine the association of MFS interval with various parameters. Clinicopathologic features at baseline were evaluated, and significantly shorter MFS intervals were observed in elderly patients (≥ 70 years, Fig. 3a) and those with T1 (Fig. 3e) or HG/G3 tumors (Fig. 3f). There was no obvious difference in MFS when assessing either patient sex, tumor size, or number (Fig. 3b–d). We also compared MFS among cases with different metastatic sites, mainly lung, bone and lymph nodes. Patients of lung metastasis showed a significantly longer MFS duration compared to counterparts with other metastatic sites (Fig. 3g). Metastases in bone happened earlier than in other sites by a mean of 14 months, although no statistical difference was found (Fig. 3h). Patients with versus without lymph node metastasis did not show MFS differences (Fig. 3i).

### Post-metastatic treatments and outcomes

**Fig. 4:**
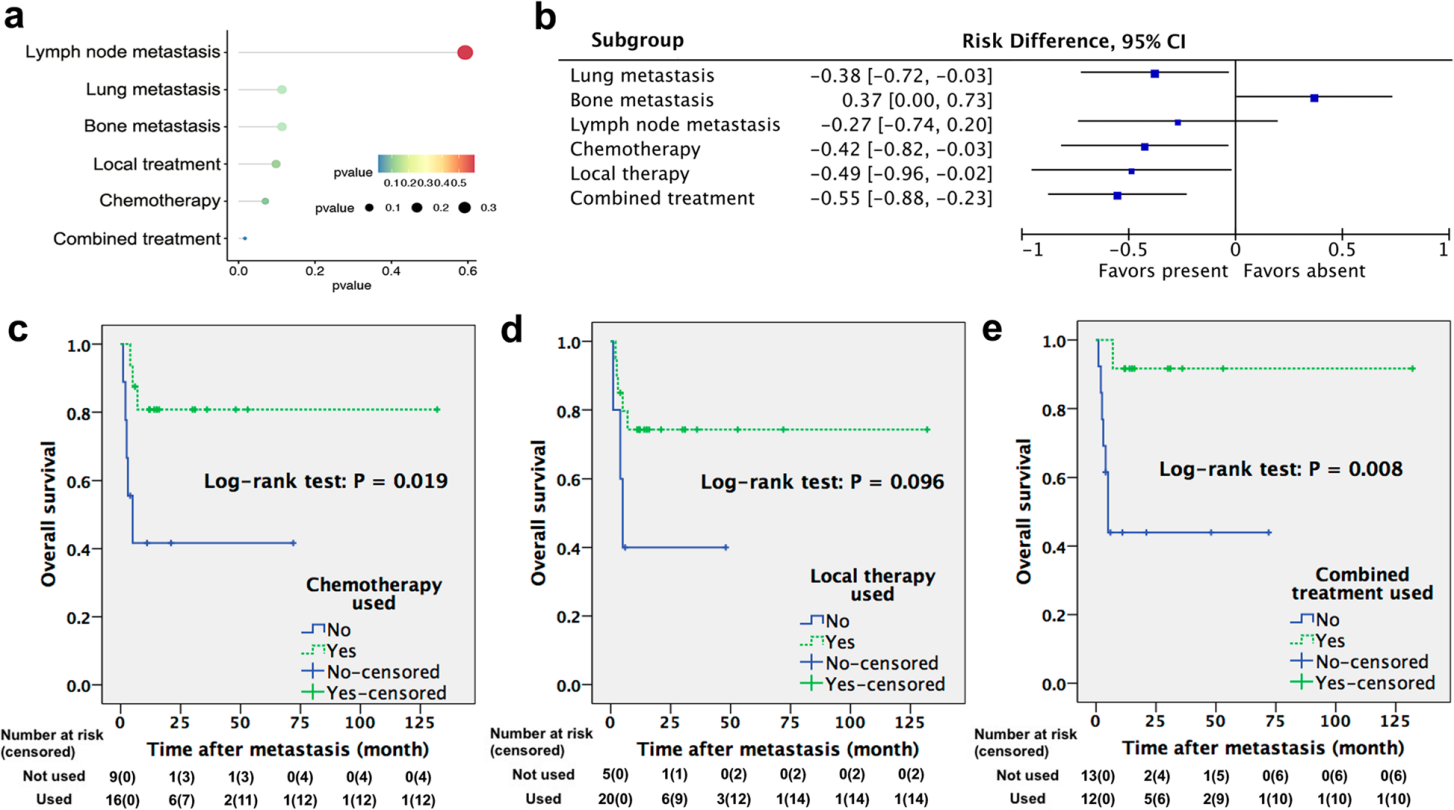
Lollipop chart (**a**) and forest plots (**b**) showing the associations of mNMIBC survival outcomes with metastatic sites and post-metastatic therapeutic approaches; post-metastatic overall survival curves stratified according to whether adopting systemic chemotherapy (**c**), local therapy (**d**), or combined treatment (**e**)

Post-metastatic treatment was performed or recorded of 33 cases. There were 20 patients administered with chemotherapy, 20 undergoing resections of metastatic lesions or organs and 10 receiving local radiotherapy. ICI was adopted for the case from our center. Survival outcomes were available for 30 patients, among whom 14 died within half a year and 15 showed various degrees of remission after at least one year (Table 2). Briefly, outcomes of these two groups were defined as unfavorable and favorable, respectively. Compared with monotherapy, combination of systemic chemotherapy with local metastasectomy or radiotherapy got obviously more favorable outcomes. Beyond that, there was no significant association of outcomes with baseline features, MFS intervals (supplementary Table S2), metastatic sites or other classifications of treatments (Fig. 4a). However, some trends were observed. More patients with lung metastasis had favorable prognosis than counterparts with non-lung metastasis, whereas bone involvement was associated with more unfavorable outcomes. As for post-metastatic treatment, both systemic chemotherapeutics and local cytoreduction were inclined to bring about favorable outcomes, and patients benefited from combined treatment unambiguously (Fig. 4b). Overall survival curves were also drawn to assess benefits from post-metastatic treatment. Chemotherapy-based approaches provided significantly prolonged survivals, especially when combined with local treatment (Fig. 4c–e).

## Discussion

NMIBC would recur or progress to MIBC, with or without pelvic lymph node metastasis. Meta-analysis of trials for recurrent NMIBC after one or more prior BCG courses showed that bladder-preserving regimens only provided a pooled response rate of 36% at 1 year [9]. Since NMIBC may behave as an aggressive malignancy, radical cystectomy is proposed for highest-risk or BCG-unresponsive cases. However, a number of such patients still insist on preserving the bladder in view of morbidities and negative impacts on quality of life by cystectomy [10]. Hurle et al. [11] conducted a single-arm study of intravesical gemcitabine in 46 BCG-unresponsive or BCG-intolerant patients, among whom 19 presented a progression to T2–T4 or extravesical lesions, including 2 suffering from metastatic disease, within the mean follow-up of 40 months. Even in above settings, principal oncological concern is mainly determined by local progression rather than distant metastasis. It is therefore an unusual occurrence for NMIBC metastasizing distantly without regional disease, namely mNMIBC. In this study, we tried to speculate risk factors predicting distant metastasis for bladder-preserving NMIBC individuals. Inherent pathologic properties of tumors may determine the unexpected outcomes. At the first occurrence, high-risk NMIBC, mainly HG/G3 or T1/CIS lesion, was found in at least 70% of all cases. Moreover, there were 30% classified into the highest-risk subgroup, including some with variant histology or LVI which implied worse prognosis than pure high-risk counterparts. Locations of primary tumors were described by some included reports, and quite a few were located at bladder neck or trigone. Previous studies addressed the association of NMIBC in this area with worse prognosis [12, 13]. Lymphatic drainage here directly proceeds to sacral and common iliac nodes, whereas lymphatics from the remainder of bladder drain to external and internal iliac nodes [12]. The anatomical feature of bladder neck adjacent to vascular bed also provides tumor cells shortcut to vascular channels [13]. Another hypothesis is about urothelial progenitor cells predominantly distributed in this area. Tumors arising from this area might contain higher proportions of stem cells and harbor aggressive biological behaviors [14].

Metastases might also develop from occult tumor spillage in operation. There were four cases of metastases in female genital system. One possibility is that voided urine during TURBT might cause surface contamination of vagina, which can be invoked as a route of malignancy spread. Bladder perforation is a complication of TURBT, as a consequence of inadvertent full-thickness bladder wall resection. Although the overall risk of extravesical tumor seeding might not increase for the whole NMIBC population, scattered cases of implantation metastases did exist [15, 16]. Bladder perforation was reported to be burdened by NMIBC progression, implying potential oncologic risk brought by this adverse event [17]. Microscopically, TURBT breaks basal membrane and opens blood and lymphatic vessels, and increased circulating tumor cells in post-TURBT peripheral blood was reported, especially when adopting conventional piecemeal TURBT technique [18, 19]. There was no bladder perforation in our cohort, but intravascular spread of tumor cells might artificially arise from the operation and lead to unexpected metastasis from formerly superficial tumor.

Identification of biomarker has progressed over decades to predict oncologic outcomes. Those related with cell proliferation, i.e., p53, ki-67, EGFR, are associated with disease progression or unfavorable cancer-specific survival [20]. Hedegaard et al. [21] described NMIBC molecular subtypes and CD44, a cancer stem cell marker, was mostly expressed in class-3 tumors with basal-like MIBC features. CD44 was also applied for IHC-based phenotypes of T1 tumors and associated with worse progression-free survival [22]. Above markers were aberrantly expressed in primary tumors of some cases, and combination of them may provide more information to forecast mNMIBC. Besides, pathogenic variants were found by NGS detection in the case of our center. For example, tyrosine kinase signaling could be enhanced by PIK3CA activating mutation, FGFR3-TACC3 fusion, and EGFR amplification [23]. Protein bioactivity of CDH1-encoding E-cadherin might be impaired by the truncating mutation, leading to elevated metastatic potential [24]. Although the NGS analysis was performed using metastatic tissues, a number of somatic variants detected should be derived from primary tumor. It is reasonable to propose that NMIBC with extensive alterations of cellular regulating genes or proteins should be considered as aggressive disease, especially for T1HG tumors. In such cases, clinicians should determine whether the bladder may be preserved or should be removed in a timely manner.

Previous studies have addressed metastatic patterns of MIBC [5, 25–27]. For de novo mBC, 51–66% and 34–49% of patients have solitary and multiple organ metastases, respectively, mainly in bone, lung, lymph nodes, and liver [5, 25]. By contrast, more of metachronous metastases after radical or palliative treatment involve multiple distal organs [26, 27]. Our analysis showed most mNMIBC cases manifested as single-site metastasis, mainly in the lung, bone, and lymph nodes. Liver involvement is far from rare for mBC and provides worst survival outcomes [5]; however, it was reported in only three mNMIBC cases. Time to relapse, or MFS interval, is a survival prognostic factor for mBC following radical cystectomy [28]. MFS intervals of MIBC-dominated cohorts are around 1–2 years [26, 29]. However, most mNMIBC cases were not identified until later, approximately 3.5 years on average after initial TURBT. It can be inferred that mNMIBC is less malignant than “conventional” mBC, thus making mNMIBC more concealed. In this study, MFS intervals of mNMIBC cases could be influenced by patient age and pathologic features of primary lesions, and tumors with variant histology or LVI appeared to metastasize earlier. Significantly longer MFS interval was also found in patients with lung metastasis whereas relatively shorter in those with bone involvement. Lung metastasis may provide better oncologic outcomes than bone metastasis in bladder cancer [30]. This trend was observed in our cohort and conformed to the MFS difference among site-specific metastases. Anyway, our findings emphasize necessities of extra-pelvic evaluations in NMIBC surveillance, even years after initial treatment. Recently, urine-based molecular or genetic markers have been facilitating the non-invasive and precise detection of bladder cancer, especially represented by mRNA-marker Xpert test and urinary tumor DNA assay [31–34]. Likewise, plasma-based liquid biopsy using circulating tumor DNA (ctDNA) profiling may forecast mBC or mNMIBC preceding formation of radiologically significant metastases. Moreover, mutation concordance between ctDNA and matched malignant tissue is over 80% for mBC, enabling benchmarking of proposed clinical biomarkers [33].

mNMIBC was basically treated following guidelines for mBC. Platinum-containing chemotherapy should be offered as the first-line option. Case of our center showed resistance to the regimen after initial response, and ICI further retrieved favorable outcomes. Results of genomic testing, i.e., high TMB, CHEK2 mutation, predicted the response to immunotherapy [35]. Local IMRT was also performed. Radiotherapy can not only induce tumor apoptosis directly but also promote immune responses, which are often negated by immunosuppression within the tumor microenvironment. ICI therapy may reverse the localized immunosuppression and enhance radiotherapy-induced anti-tumor immunity. Combination of ICI with radiotherapy will both improve the extent of local control and provide systemic abscopal effects, thus increasing response rates in those with metastatic disease [36]. In our study, some mNMIBC cases received metastasectomies, nearly half of which were resections of pulmonary lesions. Previous anecdotal reports of pulmonary resection as part of multimodal treatment suggested improved survival in selected lung-metastatic patients. Those with multiple-site metastases may not benefit, whereas small volume metastases and use of perioperative chemotherapy are associated with favorable response [37]. Other evidences on metastasectomies suggest that cure is possible in a minority of patients with solitary metastasis, but bone and liver belong to suboptimal sites [38]. Since the role of metastasectomy is still controversial, further studies are needed to identify indications for metastatic tumor debulking by surgery or non-surgical approaches, like IMRT. Local cytoreduction should be accompanied by systemic treatment, and the oligometastatic, indolent nature of mNMIBC may make these patients better candidates for the multimodal therapies.

Our study is not devoid of limitations. The first came from the approach which extracted data from cases from different centers. Publication bias made a dent in the representativeness of this mixed mNMIBC cohort. There are diversities in surgical operations and pathologic examinations among cases from literature. Secondly, there existed incompleteness in data of clinicopathologic and therapeutic information. Besides, included cases were reported across over 30 years and variability in technical approaches might affect the comparability. The long-time span of case inclusion would also result in bias for survival analyses. Despite above limitations, this study shows the uniqueness. mNMIBC cases are recognized based on long-term precise follow-up and could not be identified from database like SEER currently. Characteristics of mNMIBC have been initially extrapolated from our collective analysis, and more relevant details should be addressed by further well-designed studies.

## Conclusion

Distant metastasis without regional progression is rare in NMIBC, but potentially occurs in bladder-preserving cases with high- or highest-risk clinicopathology or extensive alterations of cellular regulating molecules. Initially, systemic staging and extended follow-up incorporating extra-pelvic imaging or even blood-based markers might be considered among such patients. Compared with conventional mBC, mNMIBC usually metastasizes later and manifests as solitary distant lesion, mainly in the lung, bone, and lymph nodes. Survival outcomes of mNMIBC would be influenced by metastatic sites as well as therapeutic approaches, and systemic chemo-or immunotherapy combined with local cytoreduction of oligometastatic lesion may render intermediate- to long-term survival in selected patients. Further studies are necessary to elucidate the detailed characteristics and metastatic mechanisms of mNMIBC as well as seek for optimal treatment for prolonged survival benefits.

## Supplementary Information

**Additional file 1: Table S1**. Gene list of next-generation sequencing for the case of our center. **Table S2**. Comparison of baseline clinicopathologic features and metastatic patterns between subgroups with favorable and unfavorable outcomes.

## Data Availability

The datasets used and/or analyzed during the current study are available from the corresponding author on reasonable request.

## Abbreviations

BCG: Bacillus Calmette-Guérin
CIS: Carcinoma in situ
ctDNA: Circulating tumor DNA
EGFR: Epidermal growth factor receptor
HG: High grade
ICI: Immune checkpoint inhibition
IHC: Immunohistochemistry
IMRT: Intensity-modulated radiotherapy
LG: Low grade
LVI: Lymphovascular invasion
mBC: Metastatic bladder cancer
MFS: Metastasis-free survival
MIBC: Muscle invasive bladder cancer
mNMIBC: Metastatic non-muscle invasive bladder cancer
NGS: Next-generation sequencing
NMIBC: Non-muscle invasive bladder cancer
TMB: Tumor mutational burden
TURBT: Transurethral resection of bladder tumor

## References

[1] Sung H, Ferlay J, Siegel RL, Laversanne M, Soerjomataram I, Jemal A, Bray F (2021). Global Cancer Statistics 2020: GLOBOCAN estimates of incidence and mortality worldwide for 36 cancers in 185 countries. CA Cancer J Clin.

[2] Bai Y, Liu L, Yuan H, Li J, Tang Y, Pu C, Han P (2014). Safety and efficacy of transurethral laser therapy for bladder cancer: a systematic review and meta-analysis. World J Surg Oncol.

[3] Zhou Y, Zhang ZL, Luo MH, Yang H (2020). Transurethral needle electrode resection and transurethral holmium laser resection of bladder cancer. World J Surg Oncol.

[4] Zapala P, Dybowski B, Poletajew S, Bialek L, Niewczas A, Radziszewski P (2018). Clinical rationale and safety of restaging transurethral resection in indication-stratified patients with high-risk non-muscle-invasive bladder cancer. World J Surg Oncol.

[5] Dong F, Shen Y, Gao F, Xu T, Wang X, Zhang X, Zhong S, Zhang M, Chen S, Shen Z (2017). Prognostic value of site-specific metastases and therapeutic roles of surgery for patients with metastatic bladder cancer: a population-based study. Cancer Manag Res.

[6] Stein JP, Lieskovsky G, Cote R, Groshen S, Feng AC, Boyd S, Skinner E, Bochner B, Thangathurai D, Mikhail M (2001). Radical cystectomy in the treatment of invasive bladder cancer: long-term results in 1,054 patients. J Clin Oncol.

[7] Matthews PN, Madden M, Bidgood KA, Fisher C (1984). The clinicopathological features of metastatic superficial papillary bladder cancer. J Urol.

[8] Sauter G, Algaba F, Amin MB, Busch C, Cheville J, Gasser T, Grignon D, Hofstaedter F, Lopez-Beltran A, Epstein JI. Non-invasive urothelial tumours. In: Eble JN, Sauter G, Epstein JI, Sesterhenn IA, editors. World Health Organization Classification of Tumours. Pathology and Genetics of Tumors of the Urinary System and Male Genital Organs. IARC Press; Lyon: 2004. 110.

[9] Kamat AM, Lerner SP, O'Donnell M, Georgieva MV, Yang M, Inman BA, Kassouf W, Boorjian SA, Tyson MD, Kulkarni GS (2020). Evidence-based assessment of current and emerging bladder-sparing therapies for non-muscle-invasive bladder cancer after Bacillus Calmette-Guerin therapy: a systematic review and meta-analysis. Eur Urol Oncol.

[10] Shen PL, Lin ME, Hong YK, He XJ (2018). Bladder preservation approach versus radical cystectomy for high-grade non-muscle-invasive bladder cancer: a meta-analysis of cohort studies. World J Surg Oncol.

[11] Hurle R, Contieri R, Casale P, Morenghi E, Saita A, Buffi N, Lughezzani G, Colombo P, Frego N, Fasulo V (2021). Midterm follow-up (3 years) confirms and extends short-term results of intravesical gemcitabine as bladder-preserving treatment for non-muscle-invasive bladder cancer after BCG failure. Urol Oncol.

[12] Fujii Y, Fukui I, Kihara K, Tsujii T, Ishizaka K, Kageyama Y, Kawakami S, Oshima H (1998). Significance of bladder neck involvement on progression in superficial bladder cancer. Eur Urol.

[13] Segal R, Yafi FA, Brimo F, Tanguay S, Aprikian A, Kassouf W (2012). Prognostic factors and outcome in patients with T1 high-grade bladder cancer: can we identify patients for early cystectomy?. BJU Int.

[14] Nguyen MM, Lieu DK, deGraffenried LA, Isseroff RR, Kurzrock EA (2007). Urothelial progenitor cells: regional differences in the rat bladder. Cell Prolif.

[15] Mydlo JH, Weinstein R, Shah S, Solliday M, Macchia RJ (1999). Long-term consequences from bladder perforation and/or violation in the presence of transitional cell carcinoma: results of a small series and a review of the literature. J Urol.

[16] Cusano A, Murphy G, Haddock P, Wagner J (2014). Tumour seeding as a result of intraperitoneal perforation during transurethral resection of non-muscle invasive bladder cancer. BMJ Case Rep.

[17] Comploj E, Dechet CB, Mian M, Trenti E, Palermo S, Lodde M, Mian C, Ambrosini-Spaltro A, Horninger W, Pycha A (2014). Perforation during TUR of bladder tumours influences the natural history of superficial bladder cancer. World J Urol.

[18] Nayyar R, Saini S, Sharma A, Kurra S, Dogra PN (2021). Systemic dissemination of tumor cells during transurethral resection in patients with bladder tumor and its clinical relevance: a follow up study. Urol Oncol.

[19] Huang H, Wang T, Ahmed MG, Zhu L, Yang C, Li W, Wu Z, Wang X, Zhang K, Xing J (2020). Retrograde en bloc resection for non-muscle invasive bladder tumor can reduce the risk of seeding cancer cells into the peripheral circulation. World J Surg Oncol.

[20] Cheng L, Davison DD, Adams J, Lopez-Beltran A, Wang L, Montironi R, Zhang S (2014). Biomarkers in bladder cancer: translational and clinical implications. Crit Rev Oncol Hematol.

[21] Hedegaard J, Lamy P, Nordentoft I, Algaba F, Hoyer S, Ulhoi BP, Vang S, Reinert T, Hermann GG, Mogensen K (2016). Comprehensive transcriptional analysis of early-stage urothelial carcinoma. Cancer Cell.

[22] Lu J, Zhang Y, Wu C, Chu C, Liu Z, Cao Y (2021). Impact of immunohistochemistry-based molecular subtype on predicting chemotherapy response and survival in patients with T1 stage bladder cancer after bladder-preserving treatment. Jpn J Clin Oncol.

[23] Christensen E, Birkenkamp-Demtroder K, Nordentoft I, Hoyer S, van der Keur K, van Kessel K, Zwarthoff E, Agerbaek M, Orntoft TF, Jensen JB, Dyrskjot L (2017). Liquid biopsy analysis of FGFR3 and PIK3CA hotspot mutations for disease surveillance in bladder cancer. Eur Urol.

[24] Bryan RT, Tselepis C (2010). Cadherin switching and bladder cancer. J Urol.

[25] Rosiello G, Palumbo C, Deuker M, Stolzenbach LF, Martin T, Tian Z, Gallina A, Montorsi F, Black P, Kassouf W (2021). Sex- and age-related differences in the distribution of bladder cancer metastases. Jpn J Clin Oncol.

[26] Shinagare AB, Ramaiya NH, Jagannathan JP, Fennessy FM, Taplin ME, Van den Abbeele AD (2011). Metastatic pattern of bladder cancer: correlation with the characteristics of the primary tumor. AJR Am J Roentgenol.

[27] Mason J, Hasnain Z, Miranda G, Gill K, Djaladat H, Desai M, Newton PK, Gill IS, Kuhn P (2021). Prediction of metastatic patterns in bladder cancer: spatiotemporal progression and development of a novel, web-based platform for clinical utility. Eur Urol Open Sci.

[28] Kluth LA, Xylinas E, Rieken M, Kent M, Ikeda M, Matsumoto K, Hagiwara M, Kikuchi E, Bing MT, Gupta A (2015). Prognostic model for predicting survival in patients with disease recurrence following radical cystectomy. Eur Urol Focus.

[29] Morera DS, Hasanali SL, Belew D, Ghosh S, Klaassen Z, Jordan AR, Wang J, Terris MK, Bollag RJ, Merseburger AS (2020). Clinical parameters outperform molecular subtypes for predicting outcome in bladder cancer: results from multiple cohorts, including TCGA. J Urol.

[30] Shou J, Zhang Q, Zhang D (2021). The prognostic effect of metastasis patterns on overall survival in patients with distant metastatic bladder cancer: a SEER population-based analysis. World J Urol.

[31] Hurle R, Casale P, Saita A, Colombo P, Elefante GM, Lughezzani G, Fasulo V, Paciotti M, Domanico L, Bevilacqua G (2020). Clinical performance of Xpert Bladder Cancer (BC) Monitor, a mRNA-based urine test, in active surveillance (AS) patients with recurrent non-muscle-invasive bladder cancer (NMIBC): results from the Bladder Cancer Italian Active Surveillance (BIAS) project. World J Urol.

[32] Fasulo V, Paciotti M, Lazzeri M, Contieri R, Casale P, Saita A, Lughezzani G, Diana P, Frego N, Avolio PP (2022). Xpert bladder cancer monitor may avoid cystoscopies in patients under "active surveillance" for recurrent bladder cancer (BIAS Project): longitudinal cohort study. Front Oncol.

[33] Zhang R, Zang J, Xie F, Zhang Y, Wang Y, Jing Y, Zhang Y, Chen Z, Shahatiaili A, Cai MC (2021). Urinary molecular pathology for patients with newly diagnosed urothelial bladder cancer. J Urol.

[34] Dong F, Shen Y, Xu T, Wang X, Gao F, Zhong S, Chen S, Shen Z (2018). Effectiveness of urine fibronectin as a non-invasive diagnostic biomarker in bladder cancer patients: a systematic review and meta-analysis. World J Surg Oncol.

[35] Teo MY, Seier K, Ostrovnaya I, Regazzi AM, Kania BE, Moran MM, Cipolla CK, Bluth MJ, Chaim J, Al-Ahmadie H (2018). Alterations in DNA damage response and repair genes as potential marker of clinical benefit from PD-1/PD-L1 blockade in advanced urothelial cancers. J Clin Oncol.

[36] Walshaw RC, Honeychurch J, Illidge TM, Choudhury A (2018). The anti-PD-1 era - an opportunity to enhance radiotherapy for patients with bladder cancer. Nat Rev Urol.

[37] Abufaraj M, Dalbagni G, Daneshmand S, Horenblas S, Kamat AM, Kanzaki R, Zlotta AR, Shariat SF (2018). The role of surgery in metastatic bladder cancer: a systematic review. Eur Urol.

[38] Horwich A, Babjuk M, Bellmunt J, Bruins HM, De Reijke TM, De Santis M, Gillessen S, James N, Maclennan S, Palou J (2019). EAU-ESMO consensus statements on the management of advanced and variant bladder cancer-an international collaborative multi-stakeholder effort: under the auspices of the EAU and ESMO Guidelines Committeesdagger. Ann Oncol.

